# Odontological Society of Pennsylvania

**Published:** 1889-07

**Authors:** 


Subject passed.

---

### ODONTOLOGICAL SOCIETY OF PENNSYLVANIA.

THE regular meeting of the Odontological Society of Pennsylvania was held Saturday evening, April 6, 1889, at the hall, Arch and Thirteenth Streets. President Kirk in the chair.

Dr. Kirk introduced the subject of perforation of the antrum in implantation, and possible pathological results.

*Dr. Kirk.*—At the February meeting, when Dr. Ottolengui read his paper, I was compelled to leave, and I feel that I did not get the full benefit of what was said. As the subject of the antrum in its treatment is of importance to all of us, and as I have had some experience in implantation, I want to speak further upon the subject. Dr. Ottolengui reported a case where the antrum was accidentally perforated. An excessive hemorrhage followed, and, in order to stop this, he inserted the tooth, and allowed the patient to remain with the tooth in position for ten days. Symptoms of septicæmia set in, and the patient was in a critical condition for some time. The case was treated by a physician in connection with Dr. Ottolengui, and she was restored to health in due time.

The inference that Dr. Ottolengui drew, or that was drawn by those present, was that the operation of implantation was directly chargeable for the unhappy result that followed. In my remarks on the paper, which were hastily made, I tried to bring out the point that the operation of implantation in that case was not, *a priori*, to be charged with those unpleasant results, for the reason that he had a hemorrhagic disease to deal with, and the antrum filled with blood, which was allowed to remain there and decompose, and set up the attack of septicæmia which followed. I took the ground that such a result might have followed the extraction of a tooth, or any procedure that would expose the antrum. I further said that I did not believe that the mere perforation of a healthy antrum by an instrument during the operation of implantation is a dangerous proceeding. My reason for this is based upon these points. The antrum, as you all know, is lined by mucous membrane, which is contiguous with that which lines the oral cavity and naso-pharynx. It is not closed, but is open to contact of air, the same as any of the respiratory passages are; so that it is not to be classed with the closed cavities of the body, where the inflammation may arise from the sudden ingress of air. It already exists there, and the catarrhal inflammation of the lining membrane of that cavity is not to be looked upon as a serious matter at all. I think we have it very much more frequently than we suppose. I can recall half a dozen cases where, passing up the canal of bicuspid teeth, the broach has passed beyond the apex and entered the antrum. Now, in such cases as that, when inflammation of the apical end of the bicuspid of a suppurative character exists, there is no doubt that the discharge that flows into the antrum is local in its expression, and probably is never noticed.

My attention was called to it when a gentleman came to this city from Scranton, and I was asked to extract what I called a reasonably good six-year molar. Dr. Harrison Allen said, "I want you to extract that tooth, because I have determined it is the cause of an antral catarrh. I had my doubts about the necessity of extracting it. If I had no knowledge of the nasal difficulty, I would not have done it. I did as he asked me to do. In passing a broach through the socket on the palatine root, I found it readily passed into the antrum. He asked me to make a much larger opening, which I did with a drill that was spear-pointed and almost three-sixteenths of an inch in width, and he proceeded to wash the antral cavity. The patient had suffered for ten years with nasal catarrh. Dr. Allen asked him to return the next Sunday, one week later, to have the treatment renewed. He received a letter two weeks later, the man saying he did not come, as requested, because the catarrh was entirely well, without any trouble. Here was a case of alveolar abscess resulting from the apex of the palatine root, where the discharge was through the antrum and out through the nose,—one of those cases that frequently occur. It was a case that was confined to the apex of the root, and the mucous lining of the antrum was not involved. It is very probable that the inflammation set up at the denuded apex of the root extended to the entire lining membrane of the antrum. There can be no doubt that the proper treatment in such cases is to remove the cause of the trouble and extract the root, or, if possible, remove the cause of the abscess, and, after removing it, treat the mucous lining of the antrum with antiseptics and sedatives.

The antrum affords a good lodging-place for vitiating secretions. Where they are irritating and thrown off, it becomes bad, because the inflammation goes on to the formation of necrosis of the interlining bone; but, in all cases where the disease is non-malignant and purely of a chronic inflammatory character, get access to the antrum in all its parts, and treat it by washing out with proper medicines. I wish to have the thought relating to Dr. Ottolengui's operation and its possible pathological effect upon the antrum confined to cases of a non-malignant character. I do not see why the perforation of a healthy antrum should result in the slightest difficulty. I have had more experience in this. I have reported in the current number of the *Cosmos* where I have had the antrum opened twice, and so far as to get it healed: there was difficulty in keeping it from healing. The first was taking some tissue from the floor of the antrum,—made a one-fourth-inch opening, and union took place in three or four weeks afterwards. I have never yet failed where I have tried to get it closed. The man died from tuberculosis before it got well.

Now, if there is a danger in opening the antrum, I would like to have it brought out. It seems to me that too much stress is laid upon opening into that cavity, and if any gentleman knows where an injury has occurred I would be very glad to hear from him.

*Dr. James Truman.*—I do not agree with Dr. Kirk that there is no danger in opening into the antrum. I have had some experience in that direction, and I have never been able to close up readily the artificial opening. I have had several cases where it was impossible to close it up, and it continued for several years, always annoying to the patient. It seems to me that there is a practical difficulty in the way. If you make an air-hole into the antrum of, say, one-fourth of an inch, it assumes different proportions, and I do not believe that new bone will be developed to close up that opening. It occupies, in my view, very much the same relation as an alveolar abscess, although, as a secondary abscess opening into the soft tissues, it is not liable to give pain. If it is simply covered up with a development of soft tissue, without a closure, as I understand closure, there is a difficulty in keeping food from getting into the antrum. I have had to make plates in order to guard against this; otherwise constant syringing would be required.

The objection I made the other evening was simply in the implantation of teeth, as the operator might readily enter the antrum.

If this is going to happen often, then it is time to sound a warning note to prevent the careless operations of novices. I have no doubt Dr. Kirk's cases are all true, but he may get hold of one where there is some systemic difficulty that he is not aware of, and the irritation set up in the living membrane may become chronic. Possibly his cases may have all been healthy systemically, but this cannot always be.

*Dr. Faught.*—I am very much interested in the possible pathological results of implantation. One of the retarding features to the practice is, possibly, the complication of the antrum with certain relationships. I cannot agree with the statement of Dr. Kirk in reference to the communication of the antrum with the outside air. The atmosphere has not the same free ingress and egress to this cavity that it has to the other portions of the respiratory tract. Then, again, we must always remember the relation which the antrum bears to the immediately adjacent tissues,—the orbit of the eye, the frontal sinus, etc.,—for these relationships make it extremely important how we act. I agree with Dr. Truman that there may be trouble from antral relationships. I have had some experience with antral cases. The openings into it have been usually the result of extraction, and, while they are not serious, I rather dislike to approach them, knowing the trouble of keeping the cavity clean. To my mind, the greatest difficulty in antral cases does not arise from contact of air so much as from the contact of other foreign substances. In treatment, the opening is often unduly enlarged by careless treatment. I do not think we should make an opening as large as mentioned by Dr. Kirk, but you must have it large enough to cleanse the cavity. The quicker you can bring the tissues into position and induce healing, the better it seems to me to be. In the several cases I have had in difficult positions, I never had any trouble in getting them to heal.

*Dr. Head.*—I think Dr. Kirk meant to say that it is not dangerous to perforate the antrum for the purpose of implanting a tooth. If that is so, Dr. Truman's and Dr. Faught's objections to it have no good grounds, for the reason that the tooth, when implanted, grows to the adjacent tissues. The tooth cannot give rise to alveolar abscess; because the apex is filled with gold, it is impossible for any material to enter into the canal which could give rise to inflammation afterwards; therefore, if Dr. Kirk makes a hole in the floor of the antrum for the purpose of implanting a tooth, he immediately afterwards fills that hole with a substance which, by growing to the adjacent tissues, will act as a perfect plug.

*Dr. Register.*—In my judgment the antrum is so placed anatomically that there should be very little fear of penetrating it. When it does happen in a healthy mouth, I can see no reason why serious consequences should follow the operation. The antrum of Highmore is nothing more than a sinus for the purpose of producing lightness of bony tissue, and in the cavity is reflected a mucous lining, the same as through the internal parts of the body. It is so formed that the mucus secretions pass out with very little obstruction except in some catarrhal troubles, and in these there is sometimes a foreign accumulation. My only apprehension, where the antrum is perforated, would be in the apex of the root of the tooth passing into it, and thus be a source of irritation that would in time cause nature to throw out the tooth; it would prevent the natural processes from absorbing the pericementum in a regular way, it being an action which is followed by a reaction. With few exceptions, I think there is very little danger in entering the antrum, certainly not enough to prevent any one from performing the operation.

*Dr. Tees.*—During a practice of many years I have never yet met with a disease of the antrum, and for this reason my views coincide with Dr. Kirk's,—that it is hardly possible that inflammation could be set up in that cavity from merely piercing it with a drill. The many cases of extraction of diseased molars would frequently bring to our attention troubles in this cavity if inflammation so easily supervened.

*Dr. Guilford.*—I am much pleased with the paper, and also with Dr. Truman's remarks. I am happy to say that, not having implanted any teeth, I have never perforated the antrum in that way.

I think there are some here who would be glad to talk upon the subject of abscesses. Each one has had his quota of cases, and I have had some failures. I think that possibly some word regarding these might instruct others and prevent their having similar results. One failure I met with happened not long ago. I was in New York City and attended the meeting of the Odontological Society, when a paper upon diseases of the antrum was read by a gentleman from Baltimore. He recommended that the tooth should not necessarily be extracted for the purpose of gaining access to the antrum, but that it should be penetrated by drilling through the alveolar septum, between the teeth. The method was approved by Dr. Atkinson and others. In my next case I tried drilling up through the alveolar process into the antrum. I failed, because the opening was not large enough to relieve the antrum of its contents. I do not think we have room enough to make the opening sufficiently large. Another mistake is injecting into that cavity liquids that are too strong,—such preparations as chloride or sulphate of zinc, used in strength. I have had the best success with a five-per-cent. solution of carbolic acid, which is all the acid that water will take up; also with sulphate of zinc, five grains to the ounce. Have the hole large enough to treat, and do it with mild remedies in weak solution, and repeat it every day or two. In the early days of my practice, a gentleman in good health presented himself for treatment. I entered the antrum through the socket of one of the molars, and each day injected a five-per-cent. solution of carbolic acid. A cure was effected in two weeks' time. What I want to impress is the use of mild remedies. There is such a thing as stimulation, and such a thing as over-medication and irritation.

*Dr. Tees.*—Have you met with many cases of diseased antrum outside of the clinics of the dental college?

*Dr. Guilford.*—I have had in my office practice, extending back over twenty years, about eight or ten cases.

*Dr. Faught.*—I am glad that Dr. Guilford mentioned this point, for I believe that in antral trouble there is danger of over-medication. In holding back the tissues I like a straight instrument, and one point to remember is to have it perfectly smooth. You do not want a sharp, penetrating instrument, but such an one as comes in Dr. Flagg's filling set No. 5. This you can pass through the opening and with it gently push away the tissues and enlarge the opening, producing no irritation, and then wash out the cavity with a syringe. I certainly believe that nitrate of silver and many other forms of medicaments recommended in the books do more harm than good. A prescription I like very much is listerine, diluted. Also, a weak solution of Pond's extract has been a pleasing remedy to me,—on one occasion, Pond's extract and a very weak solution of phenol sodique mixed.

*Dr. James Truman.*—The antrum is not a perfectly smooth cavity inside. If you will examine a number of skulls you will agree with me that it is almost impossible to drain it, no matter how you make the hole. There are ridges there that prevent it being thoroughly emptied. If you syringe in strong solutions, you will have them remaining there, and it is likely to be a source of constant trouble. I agree with the gentleman that it is poor practice to use strong solutions.

*Dr. Place.*—I have had but one experience in opening into the antrum, and that was a short time ago. I was called in to see a gentleman who was very low with consumption. He had had a six-year molar extracted to relieve him of an abscess. The dentist, after extracting the tooth, had bored through the process into the antrum, and, as a result, produced an opening between the molar and bicuspid one-quarter inch in width, from which issued a very offensive smell. When I first saw it he had it stuffed full of cotton. I suppose in such a case there is not much to do but to keep it properly drained and treat antiseptically; but it occurred to me that the dentist, after extraction, might have removed too much of the process, and by reason of the patient's anæmic condition could not get a healthy granulation to heal it.

*Dr. Register.*—Regarding the cleansing of the antrum, I have had several of these cases, and the difference in result between the use of the syringe and the atomizer in applying medicinal agents is so much in favor of the atomizer that I now recommend it to all of these cases.

As Dr. Truman rightly puts it, the interior of the antrum is not a smooth cavity: instead of its being regular in shape, it is rather the opposite, and it is impossible in treatment to properly cleanse that cavity by using a simple syringe and applying a very little force in that way; but in the use of the atomizer, with great force back of it, the air being under pressure, and using the medicinal preparations in a weak state, it is easy to cleanse it of all accumulations, which, by decomposing, would possibly lead to necrosis. I have used it in three or four cases, and the results were satisfactory in the extreme.

*Dr. Head.*—Does the gentleman sterilize the antrum?

*Dr. Register.*—I only use the liquid mixed up in the atomization with the air.

*Dr. Kirk.*—The one point of interest to me in this matter is that formulated by Dr. Head, who said, "If Dr. Kirk makes a hole in the antrum for the purpose of implanting a tooth." That is the text I want to preach on. I do nothing of the sort. In all the operations I have performed, I have never perforated the antrum, to the best of my knowledge. I take special care to prevent it. I measure the depth of the drill, and afterwards use the reamers. If a perforation has taken place, then it is the size of the first drill I use. I do not consider a perforation of that size a dangerous proceeding, because the cavity is immediately plugged up by a sterilized tooth.

Cases of antral catarrh are more frequent than we have any idea of. People are going through life with what they suppose a mild form of catarrh, discharging from the nostril, and it may scarcely be sufficient to excite their notice. I have seen a number of cases depending upon abscessed condition of the roots of some teeth, which were in close relation to the antrum; and it has become a practice when treating these cases to ask, "Have you any nasal discharge on that side?" And I have sometimes been told that they had; they believed it was a nasal catarrh. There is a case of alveolar abscess I have now,—a second bicuspid that came to me in the last stages of suppurative inflammation. The face swelled up, and I applied remedies to the gum over the apex of the root. The swelling afterwards went down. I think time will show a discharge into the antral cavity. Where we have cases of abscess of the second bicuspid or palatine root of the sixth-year molar, and pain suddenly ceases, I think, in a majority of cases, you will find the discharge has gone into the antrum. I had a case of antral disease where all the treatment failed. A more thorough exploration of the cavity revealed a growth of tissue, so placed that it was impossible to introduce medicinal preparations properly. I broke down that wall, and then it got well.

Regarding such strong remedies as chloride and sulphate of zinc, you can use them diluted without producing destructive inflammation, a solution of two or three grains to the ounce not being dangerous.

In regard to the character of opening made into the antrum for exploring purposes, nearly all cases of antral trouble I have had have been where there was one or two teeth missing. The sort of space I would make would be on the buccal side of the ridge, up pretty high, and through the soft tissues in a way that it would form a flap for healing afterwards. I have never had any difficulty in healing.

